# Serum biomarkers to predict clinical response in proof-of-concept trials in spondyloarthritis

**DOI:** 10.1186/1479-5876-9-S2-P37

**Published:** 2011-11-23

**Authors:** Maureen Turina, Nataliya Yeremenko, Jacqueline Paramarta, Bernard Vandooren, Paul Peter Tak, Leen De Rycke, Dominique Baeten

**Affiliations:** 1 Dept. of Clinical Immunology and Rheumatology, University of Amsterdam, The Netherlands

## Background

With TNF-blockers availability for spondyloarthritis (SpA), evaluation of new drugs requires quick “go/no go” signals in small scale, short term proof-of-concept (PoC) trials. Biomarkers complementing clinical evaluations may help reducing length and size of these PoCs. We aimed to identify and validate serum biomarkers to predict clinical response at group level in these trials.

## Methods

Matrix metalloproteinase-3 (MMP-3), Pentraxin-3 (PTX-3), high sensitive C-reactive protein (hs-CRP), calprotectin, Interleukin-6 (IL-6), Vascular Endothelial Growth Factor (VEGF), and alpha-2-macroglobulin (alpha-2-MG) were selected as biomarkers [1-3]. Serum levels were determined by ELISA in healthy controls (n=20**)** and at week 0 and 2 in SpA patients treated with infliximab (5 mg/kg; week 0, 2, and 6) (n=18) or placebo (n=19). Patient and physician global assessment of disease activity and BASDAI were evaluated at week 0 and 12.

## Results

Baseline serum levels of PTX-3, hs-CRP, calprotectin and VEGF (all p<0.001) were increased in SpA compared to healthy controls, whereas no differences were observed for IL-6 and alpha-2-MG. Clinical evaluation at week 12 showed that infliximab but not placebo decreased disease activity (p<0.005). Accordingly biomarker levels remained stable in the placebo group. In contrast, a decrease of hs-CRP (p<0.0001), calprotectin (p<0.001), and IL-6 (p =0.04) was observed two weeks after infliximab initiation, with a similar trend for MMP-3 (p=0.063). Other biomarker levels were not significantly modulated. The Standardized Response Mean (SRM), reflecting the predictive value at the group level, was high for calprotectin (SRM=1.259) and good for hs-CRP (SRM=0.746) and MMP-3 (SRM=0.521). At individual level, linear regression revealed low correlations of changes in hs-CRP (r^2^ between 0.24 and 0.36) and calprotectin (r^2^ between 0.08 and 0.19) at week 2 with clinical outcome parameters at week 12.

## Conclusion

Early changes in serum calprotectin, hs-CRP, and MMP-3 showed a good ability to predict longer term clinical response in SpA at group level. These biomarkers are currently being validated in independent PoC trials.
